# Improved methods for quantifying soil invertebrates during ecotoxicological tests: Chill comas and anesthetics

**DOI:** 10.1016/j.heliyon.2023.e12850

**Published:** 2023-01-07

**Authors:** Adrian Pang, Ariane Mayrand Nicol, Allison Rutter, Barbara Zeeb

**Affiliations:** a School of Environmental Studies, Queen’s University, Kingston, ON K7L 3N6, Canada; b Department of Chemistry and Biomolecular Sciences, University of Ottawa, ON K1N 6N5, Canada; c Department of Chem. & Chem. Eng., Royal Military College of Canada, Kingston, ON K7K 7B4, Canada

**Keywords:** Soil invertebrate, Ecotoxicology, Quantification, Chill coma, Anesthetic, Springtail

## Abstract

Soil invertebrate ecotoxicological tests are important when making informed site-management decisions. However, traditional tests are time-consuming and require quantification of high numbers of soil invertebrates burrowed beneath the surface of soil. A commonly used technique to extract invertebrates from the soil is the floatation method. Due to the movement of Collembola, and the presence of small soil particulates and bubbles on the surface of the water, automatic image analysis software may inaccurately quantify the true number of individuals present. Hence, manual counting immediately following extraction, or from images, is still the most effective method utilized for quantifying floated soil invertebrates. This study investigated three novel techniques; the use of an ice-water bath, chest freezer (−12 °C) and ethanol to temporarily immobilize groups of 35 *Folsomia candida* individuals to increase accuracy during the quantification step. Active thermography to aid automatic image analysis was also investigated. Results show that while thermoimaging did not provide a distinct advantage in differentiating soil invertebrates from soil particles, both an ice-water bath and 4.75% ethanol solution were extremely effective at temporarily immobilizing *F. candida* with no apparent ill effects. The outcome of this study will assist future soil invertebrate research by increasing the accuracy of invertebrate quantifications. In addition, as the techniques caused no mortality to the invertebrates, the same individuals remain available for continuous monitoring experiments, repeated exposure, and/or multi-generational studies.

## 1 Introduction

Soil invertebrates, such as mites and springtails, play an important role in ecosystem function and health [1,2]. They contribute to soil nutrient cycling via litter decomposition and have been found to alter fungal communities by selective grazing [3,4]. Studies have found soil invertebrates to be generally more sensitive to contaminants than plant species, hence it is important to incorporate them into ecological risk assessments [5,6]. Due to their high abundance and wide distribution range, Collembola (springtails) are good bioindicators when assessing soil quality. Additionally, springtail species such as *Folsomia candida* are easy to culture and have high reproduction rates via parthenogenesis, making them suitable model organisms [7].

Several ecotoxicology tests have been developed to assess soil quality using *F. candida*, investigating the survival, behaviour, and reproduction of this species. While behavioural tests, such as avoidance tests are rapid and can additionally inform plant habitat quality, reproduction and survival tests are used more commonly [8]. Of particular focus in this paper, are the biological test methods for springtails, published by Environment Canada (EC) [9]. Briefly, replicates of age-synchronized *F. candida* juveniles are exposed to control and contaminated soils. Following a period of 28 days, the springtails are extracted from the soil and quantified to determine any effects the contaminant(s) may have had on their survival and reproduction rates. Mortality and reproduction rates of the exposed *F. candida* individuals allow for the determination of ecotoxicological values and inform researchers of potential adverse effects due to the contaminant(s) under investigation.

A variety of methods exist to extract soil invertebrates from soil for quantification, however, the floatation method is most often used as it is simpler and more expedient than other methods such as heat extraction. Flooding soils with water and gently stirring will cause the soil invertebrates to float to the surface, allowing them to be quantified. The total number of offspring at the end of an experiment may reach up to several thousand, depending on the number of replicates and treatment groups. Hence, quantification of the small springtails (1.5–3.0 mm in length) is often extremely time-consuming and labour intensive [10].

Traditionally, extracted *F. candida* individuals are manually counted under a microscope. Taking photographs of the floated springtails allows researchers to complete the counts over several days, and to revisit the data if required. In recent years, automated image analysis plugins for software (e.g., ImageJ) have been used to measure (i.e., body size) and quantify organisms [2,11,12]. In this method, multiple photographs of the same vessel are compared to identify mobile elements, which are retained in a new image. Using the ImageJ “Analyze particles” function, the number of mobile elements can then be counted as the number of springtails. This method is most commonly used with soil invertebrates on solid media, such as plaster substrate or agar [2]. Unfortunately, the accuracy of automatic image analysis methods are hindered when the organisms cluster and/or overlap each other, causing counting errors. Clusters of springtails often occur when they are extracted using the floatation method as the springtails tend to aggregate and form raft-like structures. Additionally, bubbles and other soil particulates on the surface can be mistaken for springtails. Hence, manual counting of springtails extracted from soil via the floatation method is still the most accurate method available, despite being cumbersome and time consuming.

Manual counting of springtails either by analyzing images or during live counts can be hindered by the movement of the individuals. During live counts of large populations, the accuracy of quantification is often unreliable as it is difficult to determine if an individual that has moved was already accounted for. Taking photographs of the floated springtails may help alleviate this issue, however, photographs need to be taken when minimal Collembola movement is present, as it can cause blurring in the images. Hence, the immobilization of Collembola individuals could aid in increasing the accuracy and precision of manual quantification methods.

Several techniques have been shown to be effective for immobilizing invertebrates. Chemical agents such as isoflurane, carbon dioxide, tricaine and benzocaine have been shown to be effective anesthesia agents, but ethanol is more cost-effective and readily available [[13], [14], [15]]. Low concentration ethanol solutions (<10%) have previously been used to anesthetize some terrestrial and aquatic invertebrates but not Collembola [15–17]. The induction of chill comas to invertebrates is another simple and cost-effective method to temporarily immobilize invertebrates. Most insects exposed to cold temperatures can enter chill comas, causing temporary paralysis but this state is completely reversible [18]. This neuromuscular impairment is caused by depolarization of the muscle resting membrane potential, and hindrance to propagation of action potentials [19,20]. Depending on the exposure temperature and duration, the insects are able to recovery from the chill coma after a short period of rewarming. However, prolonged exposure can lead to death due to irreparable damage to the tissues of the exposed individuals, which needs to be avoided when conducting subsequent or multi-generational studies [19,21].

Multigenerational studies and subsequent exposure studies have been shown to be important and increasingly relevant with persistent contaminants. Standard exposure tests only assess the toxicity of parent generations, but adverse effects have been observed in subsequent generations, despite individuals being transferred to clean soils [[22], [23], [24], [25]]. Hence, the loss of individuals must be mitigated in order to preserve adequate quantities of individuals for future tests.

The aim of this study was to evaluate new techniques that can increase the efficiency of current methods for quantifying soil invertebrates extracted from soils following the floatation method, while mitigating unnecessary deaths to the soil invertebrate populations. To solve the issue of blurred images, clustering, and interaction with soil particulates, two methods to temporarily immobilize *F. candida* populations (i.e., induced chill coma and ethanol anesthesia) were investigated. Thermography was also assessed for its efficacy in differentiating between *F. candida* individuals and soil particulates to aid in automatic imaging analysis accuracy.

## 2 Methods

### 2.1 Soil invertebrates

*F. candida* were cultured using standardized techniques on ∼1 cm of plaster of Paris substrate, darkened with activated charcoal, in translucent plastic containers at the Royal Military College of Canada [9]. Cultures were maintained at 20 ± 3 °C with a photoperiod of 16 h light: 8 h dark. Cultures were transferred to a new substrate container every 1 or 2 months, or as required (e.g., due to overcrowding, excess waste, and cracked substrate). Culture containers were aerated and hydrated (as required) twice weekly and were fed activated granulated yeast once a week [9].

### 2.2 Immobilization of F. candida

#### 2.2.1 Chill coma method; using −12 °C chest freezer

**Fig. 1:**
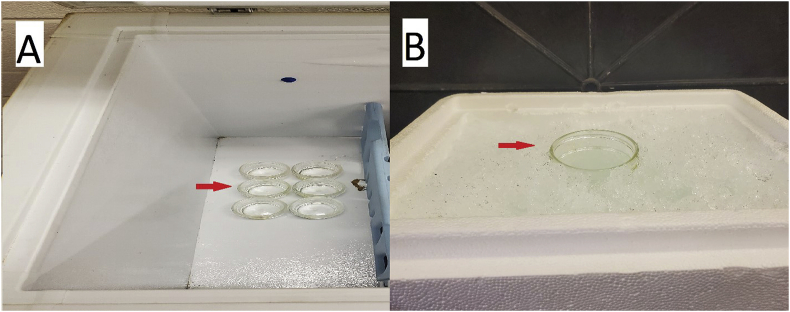
Two methods to induce chill comas to *F. candida* individuals floated in DI water. (A) Chest freezer set at −12 °C. (B) Ice-water bath within a Styrofoam cooler. Red arrows point to the arrangement of mason jars in the chest freezer and ice-water bath, respectively. (For interpretation of the references to colour in this figure legend, the reader is referred to the Web version of this article.)

In this first method, a low-suction vacuum water-based aspiration apparatus and a Leica Wild M3Z stereo microscope, at 65× magnification, was used to transfer 35 randomly-selected *F. candida* into 125 ml mason jars (6.4 cm depth and 7 cm diameter) containing 30 ml of deionized (DI) water. The mason jars containing springtails were then placed into a horizontal chest freezer at −12 °C for 5, 10, 15, or 20 min, in replicates of six (Fig. 1A). The mason jars were removed from the freezer after the specified time and the temperature of the DI water was immediately recorded. The number of individuals that entered a state of chill coma were manually quantified, transferred onto a Petri dish with plaster substrate using a small spoon and left at room temperature. Individuals were considered to be under a state of chill coma when complete cessation of movement was observed. The number of surviving springtails was recorded after a 24 h recovery period.

#### 2.2.2 Chill coma method; using ice-water bath

The second method to induce chill comas was via an ice-water bath. Using the same equipment described above, groups of 35 randomly-selected *F. candida* were transferred into 125 ml mason jars containing 30 ml of DI water, in replicates of six, and placed into an ice-water bath (Fig. 1B). The amount of time for all the individuals in each replicate to enter a chill coma, and the temperature of the DI water inside each mason jar was recorded. The individuals were then removed from the water and transferred using a small spoon into a Petri dish with plaster substrate. After a 24 h recovery period at room temperature, the surviving individuals were manually counted.

#### 2.2.3 Ethanol anesthesia method

The use of ethanol to anesthetize *F. candida* populations was investigated using a stock solution of 95% laboratory-grade ethanol diluted to 4.75% using DI water. Using the same equipment described above, 35 randomly-selected *F. candida* individuals were transferred to 125 ml mason jars and were then floated using 30 ml of the 4.75% ethanol solution, in replicates of six. The amount of time required for all the springtails to become anesthetized was recorded. Springtails were considered to be fully anesthetized when movement of the individuals completely ceased. Springtails were then removed from the ethanol solution using a small spoon, and rinsed with DI water. After a 1 h recovery period in a Petri dish containing plaster substrate at room temperature, the number of individuals to resume normal movement was recorded.

### 2.3 Thermography to differentiate F. candida from substrate

Active thermography, which employs the use of a heat source (e.g., incandescent lamp) to give the target surface minute temperature differences based on emissivity, was employed to differentiate *F. candida* and substrate material in their culturing containers. Cultures of *F. candida* were imaged under a long wavelength infrared camera (Flir A655sc) with a 640 × 480 pixel resolution that can detect temperature differences as small as 30 mK. A small amount (<1 g) of artificial soil was dispersed onto the surface of the plaster substrate to determine if the *F. candida* individuals exhibited different heat signatures than the soil.

## 3 Results and discussion

### 3.1 Chill coma of F. candida

#### 3.1.1 Using −12 °C chest freezer

When groups of 35 *F. candida* individuals floated in 30 ml DI water were exposed to −12 °C for 5 min, only 24% of individuals entered a state of chill coma, with 100% of the exposed individuals resuming normal movement after 24 h (Table 1). After 10 min of exposure, 92% of *F. candida* individuals entered a state of chill coma, and 99% of them resumed movement within 24 h at room temperature. All *F. candida* individuals entered a state of chill coma when exposed to −12 °C for 15 and 20 min, however, recovery rates of these two groups when reintroduced to room temperature for 24 h were only 38% and 40%, respectively. The mean water temperatures *F. candida* were floated in after 5, 10, 15 and 20 min in the freezer were 17 ± 0.41 °C, 10 ± 0.45 °C, 9 ± 0.50 °C and 6 ± 0.51 °C, respectively.

**Table 1:** Average (n = 6) duration, percentage of immobilized springtails and percent recovery of springtails, with 95% confidence intervals for the three different immobilization techniques used.

Treatment	Duration (min)	% Chill Coma	% Recovery
Chest freezer (−12 °C)	5	24 ± 15	100 ± 0
	10	92 ± 2.9	99 ± 1.6
	15	100 ± 1.2	38 ± 44
	20	100 ± 0	40 ± 27
Ice-water bath	9.9 ± 1.3	100	100 ± 0
Ethanol (4.75%)	12.2 ± 3.2	100	100 ± 0

Recovery was assessed after 24 h for the chest freezer and ice-water bath method, and 1 h for the ethanol treatment. Grey cells indicate values predetermined during experimental design.

Exposure to low temperatures for short durations (≤10 min) were successful in inducing chill comas with high recovery rates, however, prolonged exposure lead to high rates of mortality. This is likely caused by both indirect and direct injuries, such as membrane phase transitions, dissipation of membrane ion gradients, and/or formation of ice crystals in tissues [18]. Boiteau and MacKinley [26] determined critical temperature thresholds to induce chill comas in *F. candida* ranged from 4.15 ± 0.54 °C and 12.2 ± 1.03 °C, depending on the culturing temperature, body size and humidity. However, in their study, *F. candida* individuals were placed directly onto a clear Petri dish (i.e., without water) and chilled using a BioQuip portable chill table. Additionally, recovery from chill coma in their study was identified as the temperature one or more insects resumed movement, and the percentage of springtails to recover from chilling were not reported.

In this study, chill comas were successfully induced to *F. candida* individuals without the use of specialized equipment. It was determined that the optimal duration of exposure at −12 °C was 10 min to induce a chill coma to a majority (i.e. 92%) of floated *F. candida* individuals regardless of body size, with a recovery rate of 99% of the individuals exposed. In this study, the mean temperature of the water at which 92% of Collembola entered a state of chill coma was 10 °C, which is within the same range as employed by Boiteau and MacKinley [26] when they chilled *F. candida* individuals on petri dishes.

#### 3.1.2 Using ice-water bath

The amount of time to induce a chill coma in 100% of *F. candida* individuals floated in water among six replicates, using an ice-water bath, ranged from 8.6 to 12.0 min (Table 2). The mean time required for all individuals to become immobilized was 9.9 min (Table 1). The mean temperature of the DI water within the mason jar, at the time all movement ceased was 3.0 ± 1.0 °C. Normal activity was resumed in 100% of the individuals after they were removed from the water and placed into a Petri dish with plaster substrate for 24 h.

**Table 2:** Amount of time required to immobilize all *F. candida* individuals (n = 35), and percentage of individuals to recover from the ice-water and ethanol solution (4.75%) treatment.

Treatment	Replicate	Time (min)	Recovery (%)
Ice-water bath	A	8.60	100
	B	12.0	100
	C	9.45	100
	D	8.82	100
	E	10.3	100
	F	10.4	100
Ethanol Solution (4.75%)	A	11.6	100
	B	9.00	100
	C	9.17	100
	D	12.0	100
	E	13.5	100
	F	17.1	100

Recovery was assessed after 24 h for the ice-water bath method, and 1 h for the ethanol treatment.

The use of an ice-bath was more efficient in inducing chill comas to *F. candida*, with higher recovery rates, than the chest freezer set at −12 °C. Results suggest that −12 °C ambient temperature in the freezer is causing cold-related injuries to *F. candida* individuals when exposure durations exceeded 10 min. By monitoring the activity of *F. candida* in an ice-water bath, extra caution can be taken to mitigate any cold-related injuries. Hence, the use of an ice bath to induce chill comas in *F. candida* is recommended, as it provides 100% success rate in both inducing a chill coma and recovery of normal activity.

In this study, springtails were floated using 30 ml DI water to simulate conditions in which they are extracted from soil. Thirty ml of DI water was chosen as it is a suitable volume of water to extract springtails at the end of toxicity tests following EC's biological test methods [9]. It is important to note that, if a different volume of DI water is used to float the springtails, the duration of time required to induce chill comas to the springtails will differ from those reported here due to the change in mass of the water.

These tests were performed using groups of 35 *F. candida*, which is less than what is normally observed in 28-day reproductive tests for the species (>100 per control replicate). It is, however, unlikely that higher numbers of individuals will increase the amount of time required to induce chill comas. *Folsomia candida* are small ectotherms and any heat transfer among neighbours would be negligible. In tests where large numbers of *F. candida* are present, dilution of the floated individuals may still be required to prevent excessive overlapping/clustering of the springtails.

### 3.2 Ethanol anesthesia

Among the six replicates, the amount of time for all *F. candida* individuals to become anesthetized using a 4.75% ethanol solution, ranged between 9.0 and 17.1 min, with a mean of 12.2 min (Tables 1 and 2). After removal of the anesthetized springtails from the ethanol solution and washing with DI water, all (i.e. 100%) of the springtails recovered and resumed normal activity within an hour.

The use of ethanol to anesthetize, euthanize and/or preserve invertebrates (e.g., *Drosophila* spp., land snails and marine invertebrates) has previously been shown to be effective [16,27]. However, the use of ethanol to anesthetize Collembola has not yet been published. Where Collembola were exposed to ethanol in previous studies, it was with high concentrations (e.g., >70%) to euthanize and/or preserve the organisms, eliminating the possibility of conducting subsequent exposure studies [[29], [30], [31], [32]]. Results of this study suggests that anesthesia using a 4.75% ethanol solution is suitable in anesthetizing *F. candida* quickly.

It is important to note that recovery from cold-induced chill coma and ethanol anesthesia were recorded after different periods (i.e., 1 vs. 24 h(s) for anesthesia and chill comas, respectively). Although *F. candida* individuals are expected to recover from chill comas within a couple hours of exposure, survival was recorded 24 h after the chill comas to ensure there were no delayed mortalities due to cold-temperature related injuries.

### 3.3 Recovery from chill Comas and ethanol anesthesia

In addition to minimizing image blurring and counting errors, the immobilization of *F. candida* allows for endpoints to be recorded without injuries to the organisms. This is critical when performing studies where ongoing measurements are made during the study, and/or the organisms are needed in the future. Hence, using these techniques, continuous monitoring experiments, repeated exposure and/or multi-generational studies can be carried out without invertebrate mortality. Loss of individuals due to handling injuries (i.e., crushed by paintbrushes or excessive force during vacuuming with water aspirator) during traditional measurements lead to smaller sample sizes which may decrease the reliability of data produced in subsequent exposure tests. By temporarily immobilizing *F. candida*, the organisms can be more easily handled and transferred between test vessels without mortality and/or loss of individuals.

### 3.4 Thermal imaging to differentiate F. candida from substrate

**Fig. 2:**
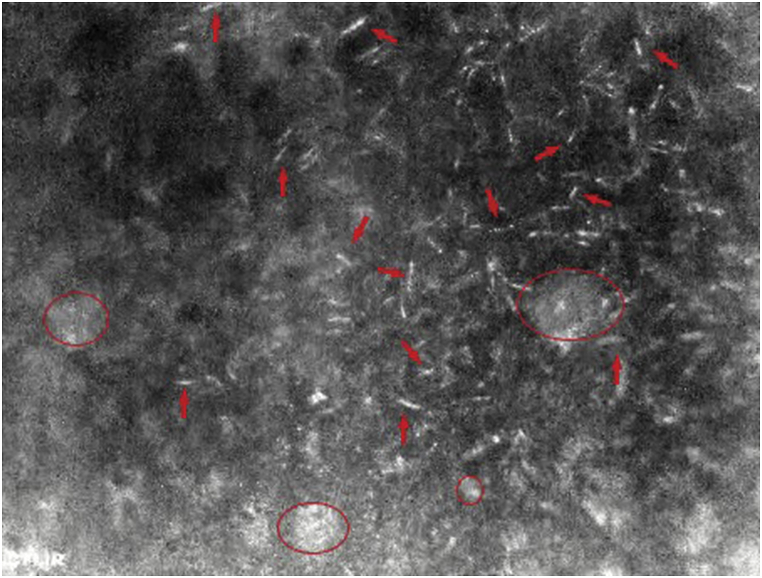
Thermographic imaging of *F. candida* colony. Image is focused on the right half. Dozens of *F. candida* individuals (red solid arrows) can be seen on top of a plaster substrate. Soil and Collembola waste (red circle) can also be seen. (For interpretation of the references to colour in this figure legend, the reader is referred to the Web version of this article.)

The *F. candida* colony was viewed using the FLIR tools live image function and was not distinguishable from the plaster substrate through thermography as they were virtually the same temperature as the substrate when using the FLIR A655sc camera alone. Using active thermography (i.e., use of an external heat source) directed at the colonies, three short videos were recorded. Active thermography allowed for the differentiation between *F. candida* individuals and the surface of plaster substrate (Fig. 2). However, in some cases, springtails where not distinguishable from soil particles and/or springtail waste (i.e., feces and shed exoskeletons) due to similar heat signatures. Additionally, *F. candida* individuals hidden under soil were not visible under the infrared light (IR) camera.

*F. candida* are strictly ectotherms and regulate their body temperatures by migrating to optimal temperatures [26]. When confined within a container, their ability to migrate to ideal temperatures is limited, and their body temperature will closely reflect that of ambient temperatures. Using an external heat source, the difference in emissivity of *F. candida* and the substrate was enough to contrast the individuals against the substrate. Although Collembola and soil can safely be assumed to have different emissivity values, due to differences in chemical composition, variable surface uniformity and waste caused certain discrepancies. The use of active thermography is unlikely to help reduce the number of erroneous counts when using automatic image analysis. Given that active thermography does not fully eliminate soil particles during imaging, the high cost of the equipment is not justified for springtail quantification using automatic image analysis software. Hence, manual counting methods may still be the most reliable and accurate technique to quantify springtails following floatation.

## 4 Conclusion

Soil invertebrates play a crucial role in ecosystems, and are important to consider when performing ecological risk assessments. Manually counting invertebrates following extraction from soil using the floatation method is a time-consuming and laborious task, especially when the populations are mobile. Here, two methods were identified to efficiently immobilize *F. candida* temporarily with 100% recovery rates (i.e., an ice-water bath and 4.75% ethanol solution). These methods using nonspecialized and easily accessible equipment have not been previously published for *F. candida*. Using these immobilization methods following the extraction of Collembola using the flotation method, manual counting immediately, or more focused pictures can be taken to ensure accurate quantification. In addition to increasing accuracy of quantification methods, temporarily immobilizing Collembola with high recovery rates may be beneficial as it allows for subsequent exposure of the same extracted springtails for chronic multi-generation studies.

## Declarations

### Author contribution

Adrian Pang: Conceptualization; Data curation; Formal Analysis; Investigation; Methodology; Validation; Resources; Software; Visualization; Writing-original draft. Ariane Mayrand Nicol: Investigation; Methodology; Writing-review & editing. Allison Rutter: Funding acquisition; Project; Supervision; Writing-review & editing. Barbara Zeeb: Funding acquisition; Project; Supervision; Writing-review & editing.

### Data availability

Data is available from the corresponding author (adrianpang94@gmail.com).

### Funding statement

This work was supported by the Natural Sciences and Engineering Research Council of Canada (NSERC Discovery Grant #RGPIN-2021-02430) to Barbara Zeeb.

## Conflict of interest

The authors declare no conflict of interest.
